# Examining Migraine as a Predictor of Benign Paroxysmal Positional Vertigo Onset, Severity, Recurrence, and Associated Falls

**DOI:** 10.7759/cureus.28278

**Published:** 2022-08-22

**Authors:** Eric K Kim, Lauren Pasquesi, Jeffrey D Sharon

**Affiliations:** 1 Otolaryngology Head and Neck Surgery, University of California San Francisco, San Francisco, USA

**Keywords:** migraine, positional vertigo, vestibular disease, migraine disorder, benign paroxysmal positional vertigo

## Abstract

Introduction: The comorbidity of migraine and benign paroxysmal positional vertigo (BPPV) is well-established, yet the impact of migraine on the BPPV phenotype remains understudied.

Methods: A retrospective analysis of patients at a tertiary dizziness/vertigo clinic diagnosed with BPPV from 2015 and 2020 was conducted. The study's primary outcomes were the age of BPPV onset, Dizziness Handicap Index (DHI), BPPV recurrence, and dizziness-related falls.

Results: In our cohort of 255 BPPV patients, 44.7% had a history of migraine. Those with migraine had an earlier age of BPPV onset than individuals without migraine (60.2 vs. 65.4, p = 0.0018). Migraineurs and non-migraineurs did not differ in their DHI (44.7 vs. 41.6, p= 0.44), recurrence rates (48.3% vs. 40.4%, p= 0.21), and falls (32.5% vs. 37.6%, p = 0.39). Among individuals with horizontal canal BPPV, a higher proportion of migraineurs experienced falls than non-migraineurs (50.0% vs. 6.3%, p = 0.02).

Conclusions: Migraineurs experience BPPV at a younger age than those without migraine. This finding suggests that migraine, which has been shown to cause inner ear damage, predisposes individuals to developing BPPV earlier. Migraine was also associated with a higher rate of falls among patients with horizontal canal BPPV, indicating that a migraine history may impact the phenotype of BPPV.

## Introduction

Benign paroxysmal positional vertigo (BPPV) is one of the most common causes of dizziness and vertigo, with an estimated lifetime prevalence of 2.4% [1]. While BPPV is, as its name suggests, benign in nature, it nevertheless causes debilitating episodes that interfere with daily living and is associated with decreased quality of life [2]. Individuals suffering from BPPV experience higher levels of anxiety and depression and report negative life events more frequently than healthy controls [3].

BPPV most commonly affects individuals in the fifth or sixth decade of their lives with a female preponderance [1]. It presents as brief episodes of vertigo lasting seconds and is triggered by changes in head movement [4], although the duration of vertiginous symptoms can widely vary. This presentation can be explained by the pathophysiology of BPPV, which involves the displacement of otoconia from the utricle. Posterior canal BPPV (PC-BPPV) is caused by floating otoconia causing endolymphatic currents in the posterior semicircular canal. Horizontal canal BPPV (HC-BPPV) with geotropic nystagmus is similarly caused by canalolithiasis in the horizontal canal [5]. HC-BPPV with ageotropic nystagmus is thought to be due to the adherence of otoconia to the cupula [6], although our previous study suggested that utricular dysfunction may also be involved [7].

The comorbidity of BPPV with other health conditions is well-documented. It is associated with a higher risk for ischemic strokes [8], diabetes [9], osteoporosis [10], and migraine [11-13]. Notably, migraine was three times more prevalent among individuals with BPPV in a retrospective study [13]. Large epidemiological studies further corroborated this relationship, as individuals with migraine had an increased risk of BPPV [11,12]. The association between BPPV and migraine has led some to postulate that migraine and vestibular dysfunction may share an underlying pathomechanism [13].

Migraine may impact the phenotype of BPPV. In an analysis of 186 patients, Faralli et al. demonstrated that migraineurs have a higher prevalence and earlier onset of BPPV and experience greater post-BPPV residual dizziness compared to those without migraine [14]. Despite the well-established link between these two conditions, there is limited literature exploring how migraine influences the clinical presentation of BPPV that either supports or disproves the article by Faralli et al. [14]. Our objective was to address this gap and analyze how migraine may predict BPPV’s onset, severity, recurrence, and dizziness-associated falls. This study was previously presented as a poster at the 2021 Vestibular-Oriented Research (VOR) Meeting on February 16, 2021.

## Materials and methods

We retrospectively reviewed consecutive adult patients diagnosed with BPPV from January 2015 to June 2020 at a tertiary referral center. Migraine history was indicated by the patient on a questionnaire. The diagnosis of BPPV was made in accordance with Barany Society guidelines by our otolaryngology practice [15]. Posterior canal BPPV (PC-BPPV) was diagnosed when a geotropic, torsional upbeat nystagmus was elicited with the Dix-Hallpike maneuver. Horizontal canal BPPV (HC-BPPV) was diagnosed when horizontal nystagmus (both geotropic and ageotropic) was observed during the supine roll. Our primary outcomes were the age at initial BPPV presentation, Dizziness Handicap Index (DHI), recurrence of BPPV after treatment, and dizziness-related falls. This study was approved by the University of California San Francisco Institutional Review Board (IRB 18-25360).

Descriptive statistics (mean, standard deviation (SD), and percentages) were used. We utilized the two-sample t-test to compare the mean ages at diagnosis and DHI scores between migraine and non-migraine groups. The Chi-square test or Fisher’s exact test was utilized to compare the rates of history of falls and recurrence. Stata (Version 16.1) (StataCorp LLC, College Station, Texas, USA) was used for statistical analysis. p-value < 0.05 was considered statistically significant.

## Results

Our cohort included 255 patients; 227 (89.0%) and 28 (11.0%) had PC-BPPV and HC-BPPV, respectively. The mean age of the cohort was 63.0 (SD: 13.4). Of all individuals, 114 (44.7%) had a history of migraine. The rates of migraine did not differ between the PC-BPPV and HC-BPPV groups (44.9% vs. 42.9%, p = 0.50). The data of the whole cohort are shown in Table 1, and subgroup analysis by BPPV type is shown in Tables 2, 3.

**Table 1:** Analysis of all patients with BPPV. *Statistically significant, p < 0.05. BPPV: benign paroxysmal positional vertigo.

	Total (N = 255)	Migraine (N = 114)	No migraine (N = 141)	p-value
Age (SD)	63.0 (13.4)	60.2 (13.0)	65.4 (13.2)	0.0018*
Dizziness Handicap Index (SD)	42.8 (25.0)	44.7 (25.0)	41.6 (25.1)	0.44
Recurrence (%)	43.9	48.3	40.4	0.21
Dizziness-related falls (%)	35.2	32.5	37.6	0.39

**Table 2:** Subgroup analysis of patients with posterior canal BPPV. *Statistically significant, p < 0.05. BPPV: benign paroxysmal positional vertigo.

	Migraine (N = 102)	No migraine (N = 125)	p-value
Age (SD)	60.3 (13.1)	65.5 (13.7)	0.0038*
Dizziness Handicap Index (SD)	46.5 (26.0)	43.3 (25.8)	0.47
Recurrence (%)	48.0	38.4	0.14
Dizziness-related falls (%)	30.4	41.6	0.08

**Table 3:** Subgroup analysis of patients with horizontal canal BPPV. *Statistically significant, p < 0.05. BPPV: benign paroxysmal positional vertigo.

	Migraine (N = 12)	No migraine (N = 16)	p-value
Age (SD)	59.2 (12.8)	64.5 (9.6)	0.22
Dizziness Handicap Index (SD)	35.4 (16.8)	27.8 (11.6)	0.24
Recurrence (%)	50.0	56.3	0.74
Dizziness-related falls (%)	50.0	6.3	0.02*

Individuals with a migraine history had an earlier onset of BPPV than those without (p = 0.0018). The mean age of BPPV diagnosis was 60.2 (SD: 13.0) and 65.4 (SD: 13.2) for those with and without migraine, respectively. In the PC-BPPV group, migraineurs experienced BPPV at an earlier age than non-migraineurs (60.3 vs. 65.5, p = 0.0038). In the HC-BPPV group, migraineurs also had an earlier age of diagnosis than non-migraineurs (59.2 vs. 64.5, p = 0.22), although the difference did not reach statistical significance.

Among 163 BPPV patients with documented DHI scores, DHI did not differ between those with and without migraine (p = 0.44). The mean DHI scores were 44.7 (SD: 25.0) and 41.6 (SD: 25.1) for 62 individuals with migraine and 101 without migraine, respectively. In the PC-BPPV group, DHIs were statistically similar between patients with and without migraine (46.5 vs. 43.3, p = 0.47). In the HC-BPPV group, the DHI was higher among migraineurs than those without migraine (35.4 vs. 27.8, p = 0.24), although the difference was statistically non-significant.

BPPV recurred in 43.9% of all patients, although there was no difference between the recurrence rate of BPPV symptoms between migraineurs and non-migraineurs (48.3% vs. 40.4%, p = 0.21). In our subgroup analysis, recurrence rates were statistically similar between those with and without a migraine history in both PC-BPPV (48.0% vs. 38.4%, p = 0.14) and HC-BPPV (50.0% vs. 56.3%, p = 0.74) groups.

Over a third of BPPV patients (35.2%) experienced a fall due to dizziness. The rates of falls did not differ between patients with and without migraine (32.5% vs. 37.6%, p = 0.39). In the PC-BPPV group, the rates of falls were statistically similar between the migraineurs and non-migraineurs (30.4% vs. 41.6%, p = 0.08). In the HC-BPPV group, a higher proportion of migraineurs experienced falls than non-migraineurs (50.0% vs. 6.3%, p = 0.02).

## Discussion

Migraine was present in almost half of all BPPV patients. Among those with PC-BPPV, migraineurs on average were younger by five years than individuals without migraine when they first experienced BPPV. A history of migraine was not associated with greater severity of functional impairment, an increased risk for dizziness-related falls, or recurrence of symptoms. However, among HC-BPPV patients, migraineurs had a higher rate of falls.

While its exact mechanism remains unclear, the association of migraine with an earlier onset of BPPV suggests that the pathophysiology of migraine predisposes one to BPPV. Ishiyama et al. proposed that migraine causes recurrent vasospasms that damage the vasculature of the inner ear, which may in turn displace the otoconia from the otolith organs [13]. This connection is further supported by the fact that repeated vasospasms cause oxidative stress, and decreased levels of superoxide dismutase (SOD), an antioxidant protein, have been observed in both migraine and BPPV [16-18]. Additionally, calcitonin gene-related peptide (CGRP), a neuroactive peptide that is released by trigeminal nerves during migraine attacks [19], may represent another pathophysiological link. As CGRP is expressed in the efferent neurons of otolith organs and semicircular canals [20], the dysregulation of CGRP in migraineurs may increase the risk of BPPV. Another consideration is that trigeminal nerve activation in migraine results in plasma extravasation in the inner ear [21], which perhaps causes hydrops that shear otoconia from otolith organs. These potentially explain the high prevalence of migraine among our BPPV patients, which is greater than what has been previously reported [22].

Our finding that individuals with migraine experience BPPV at a younger age than those without migraine further establishes a link between the two disease entities. Our subgroup analysis revealed that migraineurs were on average younger by five years at the onset of BPPV than those without a migraine history in both PC-BPPV and HC-BPPV groups, although the age difference did not reach statistical significance among HC-BPPV patients, most likely due to the small sample size of this population. Although von Brevern et al. did not observe the impact of migraine on the age of onset [1,15], Faralli et al. found a 14-year difference in the age of BPPV onset between migraineurs and non-migraineurs [14]. Notably, the mean age of onset for those with migraine in their study was 39, which is significantly younger than the known peak age of BPPV incidence [1,14]. Our results suggest that the age difference may be smaller, as the mean age of onset for both migraineurs and non-migraineurs was in the sixth decade of life.

Despite an earlier age of onset for migraineurs, a migraine history did not predict worse functional impairment. Whitney et al. reported that the mean DHI for BPPV patients was 41.6, which corresponds to a moderate level of handicap [23]. In our cohort, the DHIs of BPPV patients with and without migraine were consistent with this result. Unlike Faralli et al., who demonstrated that migraineurs with BPPV perceived a greater handicap than BPPV patients without migraine [14], our data suggest that migraine does not impact a BPPV patient’s self-perceived impairment.

Migraine was not associated with the recurrence of BPPV. In the literature, reported recurrent rates of BPPV after canalith repositioning maneuvers widely vary between 13.3% and 65% [24-28]. Faralli et al. showed that a higher proportion of migraineurs with BPPV had highly recurrent BPPV, defined as having at least four documented episodes [14]. Extending the idea that pathophysiological processes in migraine increase the risk of otoconia dislodgment, one can see how migraine can also lead to the recurrence of BPPV. However, in a study spanning over a thousand patients, Hilton et al. concluded that recurrent rates of BPPV did not differ between those with and without migraine [22]. In our study, BPPV patients with migraines had a higher recurrence rate, although the difference was not statistically significant. More studies are required to further investigate the relationship between migraine and recurrence of BPPV.

Over a third of BPPV patients experienced falls, with migraineurs with HC-BPPV experiencing a significantly higher rate of falls than HC-BPPV patients without migraine. Lawson et al. reported that 14.5% of 850 patients admitted to the falls service had a positive Dix-Hallpike test [29], signifying that BPPV accounts for a substantial burden of falls. In our cohort, migraineurs and non-migraineurs experienced similar rates of falls. When examining those with HC-BPPV specifically, however, we observed that half of the migraineurs had had falls, compared to less than 10% of non-migraineurs. While the reason for this phenomenon is unclear, this finding suggests that migraine does play a role in the manifestation and sequelae of one’s BPPV.

This study has three notable limitations. First, our analysis did not account for other risk factors of incidence and recurrence of BPPV, such as female sex, osteoporosis, diabetes mellitus, and hyperlipidemia [24]. Future works should focus on analyzing these variables to better understand how a migraine history may impact one’s age of BPPV onset. Second, we used the patients' self-report to ascertain their migraine diagnosis, which could have been a self-diagnosis or a diagnosis made by another provider. Regardless, studies have shown good agreement between self-report and the International Classification of Headache Disorders (ICHD) diagnosis of migraine [30,31], giving legitimacy to patients’ self-report of a migraine history. Third, our study was retrospective, limited to a single tertiary institution, and may not be generalizable to other settings.

## Conclusions

The relationship between migraine and BPPV has long been studied. We demonstrate that migraineurs with BPPV have an earlier age of onset than BPPV patients without migraine. There were no overall differences in the functional handicap, recurrence, or rates of falls between migraineurs and non-migraineurs. However, in our subgroup analysis of HC-BPPV patients, migraineurs had a statistically significantly higher rate of falls than individuals without migraine. These connections are likely due to a shared element in their pathophysiology and warrant further investigation in basic and clinical studies.

## References

[1] von Brevern M, Radtke A, Lezius F, Feldmann M, Ziese T, Lempert T, Neuhauser H (2007). Epidemiology of benign paroxysmal positional vertigo: a population based study. J Neurol Neurosurg Psychiatry.

[2] Lopez-Escamez JA, Gamiz MJ, Fernandez-Perez A, Gomez-Fiñana M (2005). Long-term outcome and health-related quality of life in benign paroxysmal positional vertigo. Eur Arch Otorhinolaryngol.

[3] Monzani D, Genovese E, Rovatti V, Malagoli ML, Rigatelli M, Guidetti G (2006). Life events and benign paroxysmal positional vertigo: a case-controlled study. Acta Otolaryngol.

[4] Kim JS, Zee DS (2014). Benign paroxysmal positional vertigo. N Engl J Med.

[5] Nuti D, Vannucchi P, Pagnini P (1996). Benign paroxysmal positional vertigo of the horizontal canal: a form of canalolithiasis with variable clinical features. J Vestib Res.

[6] Schuknecht HF, Ruby RR (1973). Cupulolithiasis. Adv Otorhinolaryngol.

[7] Kim EK, Pasquesi L, Steenerson KK, Otero-Millan J, Sharon JD (2022). Vestibular test results in patients with horizontal canal benign paroxysmal positional vertigo. Cureus.

[8] Kao CL, Cheng YY, Leu HB (2014). Increased risk of ischemic stroke in patients with benign paroxysmal positional vertigo: a 9-year follow-up nationwide population study in Taiwan. Front Aging Neurosci.

[9] Cohen HS, Kimball KT, Stewart MG (2004). Benign paroxysmal positional vertigo and comorbid conditions. ORL J Otorhinolaryngol Relat Spec.

[10] Parham K, Kuchel GA (2016). A geriatric perspective on benign paroxysmal positional vertigo. J Am Geriatr Soc.

[11] Kim SK, Hong SM, Park IS, Choi HG (2019). Association between migraine and benign paroxysmal positional vertigo among adults in South Korea. JAMA Otolaryngol Head Neck Surg.

[12] Chu CH, Liu CJ, Lin LY, Chen TJ, Wang SJ (2015). Migraine is associated with an increased risk for benign paroxysmal positional vertigo: a nationwide population-based study. J Headache Pain.

[13] Ishiyama A, Jacobson KM, Baloh RW (2000). Migraine and benign positional vertigo. Ann Otol Rhinol Laryngol.

[14] Faralli M, Cipriani L, Del Zompo MR, Panichi R, Calzolaro L, Ricci G (2014). Benign paroxysmal positional vertigo and migraine: analysis of 186 cases. B-ENT.

[15] von Brevern M, Bertholon P, Brandt T, Fife T, Imai T, Nuti D, Newman-Toker D (2017). Benign paroxysmal positional vertigo: diagnostic criteria consensus document of the Committee for the Classification of Vestibular Disorders of the Bárány Society. Acta Otorrinolaringol Esp (Engl Ed).

[16] Li J, Wu R, Xia B, Wang X, Xue M (2020). Serum levels of superoxide dismutases in patients with benign paroxysmal positional vertigo. Biosci Rep.

[17] Tuncel D, Tolun FI, Gokce M, Imrek S, Ekerbiçer H (2008). Oxidative stress in migraine with and without aura. Biol Trace Elem Res.

[18] Ciancarelli I, Tozzi-Ciancarelli MG, Spacca G, Di Massimo C, Carolei A (2007). Relationship between biofeedback and oxidative stress in patients with chronic migraine. Cephalalgia.

[19] Villalón CM, Olesen J (2009). The role of CGRP in the pathophysiology of migraine and efficacy of CGRP receptor antagonists as acute antimigraine drugs. Pharmacol Ther.

[20] Jones SM, Vijayakumar S, Dow SA, Holt JC, Jordan PM, Luebke AE (2018). Loss of α-calcitonin gene-related peptide (αCGRP) reduces otolith activation timing dynamics and impairs balance. Front Mol Neurosci.

[21] Vass Z, Dai CF, Steyger PS, Jancsó G, Trune DR, Nuttall AL (2004). Co-localization of the vanilloid capsaicin receptor and substance P in sensory nerve fibers innervating cochlear and vertebro-basilar arteries. Neuroscience.

[22] Hilton DB, Luryi AL, Bojrab DI (2020). Comparison of associated comorbid conditions in patients with benign paroxysmal positional vertigo with or without migraine history: a large single institution study. Am J Otolaryngol.

[23] Whitney SL, Marchetti GF, Morris LO (2005). Usefulness of the dizziness handicap inventory in the screening for benign paroxysmal positional vertigo. Otol Neurotol.

[24] Sfakianaki I, Binos P, Karkos P, Dimas GG, Psillas G (2021). Risk factors for recurrence of benign paroxysmal positional vertigo. A clinical review. J Clin Med.

[25] Pérez P, Franco V, Cuesta P, Aldama P, Alvarez MJ, Méndez JC (2012). Recurrence of benign paroxysmal positional vertigo. Otol Neurotol.

[26] Kansu L, Avci S, Yilmaz I, Ozluoglu LN (2010). Long-term follow-up of patients with posterior canal benign paroxysmal positional vertigo. Acta Otolaryngol.

[27] Rashad UM (2009). Long-term follow up after Epley's manoeuvre in patients with benign paroxysmal positional vertigo. J Laryngol Otol.

[28] Korres S, Balatsouras DG, Ferekidis E (2006). Prognosis of patients with benign paroxysmal positional vertigo treated with repositioning manoeuvres. J Laryngol Otol.

[29] Lawson J, Bamiou DE, Cohen HS, Newton J (2008). Positional vertigo in a falls service. Age Ageing.

[30] Schürks M, Buring JE, Kurth T (2009). Agreement of self-reported migraine with ICHD-II criteria in the Women's Health Study. Cephalalgia.

[31] Qiu C, Williams MA, Aurora SK, Peterlin BL, Gelaye B, Frederick IO, Enquobahrie DA (2013). Agreement of self-reported physician diagnosis of migraine with international classification of headache disorders-II migraine diagnostic criteria in a cross-sectional study of pregnant women. BMC Women's Health.

